# Optimizing Computational Parameters for Nuclear Electronic Orbital Density Functional Theory: A Benchmark Study on Proton Affinities

**DOI:** 10.1002/jcc.70082

**Published:** 2025-03-18

**Authors:** Raza Ullah Khan, Ralf Tonner‐Zech

**Affiliations:** ^1^ Wilhelm‐Ostwald‐Institut für Physikalische und Theoretische Chemie Leipzig Germany

**Keywords:** benchmarking, computational parameters, light nuclei, NEO‐DFT, nuclear quantum effects, nuclear‐electronic orbital, proton affinity

## Abstract

This study benchmarks the nuclear electronic orbital density functional theory (NEO‐DFT) method for a set of molecules that is larger than in previous studies. The focus is on proton affinity predictions to assess the influences of computational parameters. NEO‐DFT incorporates nuclear quantum effects for protons involved in protonation processes. Using a test set of 72 molecules with experimental proton affinities as reference, we evaluated various exchange‐correlation functionals, finding that B3LYP‐based functionals deliver the most accurate results. Among the tested functionals, CAM‐B3LYP performs the best with an MAD value of 6.2 kJ/mol with respect to experimental data. In NEO‐DFT, electron‐proton correlation (epc) functionals were assessed, with LDA‐type epc17‐2 yielding comparable results to the GGA‐type epc19 functional. Compared to traditional DFT (MAD value of 31.6 kJ/mol), which treats nuclei classically, NEO‐DFT provides enhanced accuracy for proton affinities when electron‐proton correlation is included. Regarding basis sets, the def2‐QZVP electronic basis set achieved the highest accuracy with an MAD value of 5.0 kJ/mol, though at a higher computational cost compared to def2‐TZVP and def2‐SVP, while nuclear basis sets showed minimal impact on proton affinity accuracy and no consistent trend. Overall, this study demonstrates NEO‐DFT's efficacy in addressing nuclear quantum effects for proton affinity predictions, providing guidance on optimal parameter selection for future NEO‐DFT applications.

## 1 Introduction

Computational chemistry usually treats nuclei like classical particles. In most cases, this is a very good approximation. However, it has emerged in recent years that nuclear quantum effects (NQE) cannot be neglected for some chemical properties, especially for hydrogen‐containing compounds. For example, the hydrogen‐bond network in water can either become stronger or weaker depending on the direction of delocalization of the proton to the bond [1, 2, 3]. The importance of NQEs has been investigated on the structure of water [3, 4, 5, 6] and on the rate of enzyme reactions [7, 8] showing that neglecting NQEs can lead to significant errors in computational simulations. We are interested in investigating the effect of having different hydrogen isotopes (H, D, T) in a chemical bond and the resulting influence on the electronic structure. Trivially, this investigation requires taking care with the quantum nature of the nuclei since, in typical quantum‐chemical investigations using classical particles, we would only be able to capture the mass effect of the nuclei on, for example, vibrational properties. This is of great relevance since the effects of substituting hydrogen (H) by deuterium (D) have been shown to have an impact on drug efficiency [9, 10, 11, 12] and on the optoelectronic characteristics of conducting polymers [13]. Similarly, isotope substitution leads to significant geometric alteration in chemical systems [14, 15, 16].

On our way to find a suitable computational description of these isotope effects, we however, have to start from investigating NQEs on hydrogen at first since not much comprehensive data are around in the literature to give guidance on how to choose a suitable computational chemistry model for these effects. We thus decided to start our investigations with the analysis of NQEs on proton affinities (PA) since this property is intricately connected to a proper description of the hydrogen atom and its bond. In future investigations, we will extend this work to hydrogen isotopes. We want to emphasize that our goal is not to provide a new method for achieving accurate PAs since there are many (wave function based) methods available which give excellent agreement with experiment. Our main focus is to use a chemically relevant and suitable property to benchmark our computational approach.

The explicit treatment of NQEs is a challenging field, and several methodological advances have been made in recent years [17, 18, 19, 20, 21, 22, 23, 24, 25]. However, most of these methods are not suitable for computational investigations of larger systems or extended sets of molecules, which is our goal. The use of the nuclear electronic orbital approach to density functional theory (NEO‐DFT) represents an efficient and accurate method that explicitly accounts for the quantum nature of both electrons and (selected) nuclei [26, 27]. Although some benchmark studies on NEO‐DFT are available, the test sets comprised only a few small molecules [28, 29] and lacked systematic benchmarking of the computational parameters.

We now use the example of proton affinity calculations to provide a test of computational parameters for NEO‐DFT. We chose a set of 72 molecules from different compound classes such as amines, amides, esters, alcohols, ethers, aldehydes, ketones, and carboxylic acids whose proton affinities are experimentally known (Table S1 in the Supporting Information) [30]. PA is a specifically suitable property for the treatment with NEO‐DFT because it captures a key energy ingredient: the change in zero‐point energy (ZPE) between the protonated and unprotonated states. By focusing on proton affinity, we address a property inherently linked to the quantum proton (and its associated properties such as nuclear densities and ZPEs which were initially considered for checking accuracy of NEO during development of electro‐proton correlation functionals), making it a good prospect for benchmarking and validating the NEO‐DFT framework.

Benchmarking studies are essential in computational chemistry as they enable the evaluation of a method's accuracy relative to experimental or high‐quality computational reference data. Extensive benchmarking has been conducted for standard DFT methods, focusing primarily on assessing the accuracy of different density functionals [31, 32, 33]. However, a second level of benchmarking is also crucial: evaluating the computational parameters within a given DFT framework for a selected functional, particularly regarding convergence based on the chosen basis set. For NEO‐DFT calculations, additional parameters require careful consideration, including the choice of nuclear basis sets and electron‐proton correlation functionals. Furthermore, it remains unclear whether selecting a density functional for the electronic problem or ensuring convergence with electronic basis sets is impacted by the shift from DFT to NEO‐DFT. To address this gap, we present a comprehensive data set focused on proton affinities, systematically examining how these parameters influence PA prediction accuracy against experimental data. These findings offer guidance for future NEO‐DFT applications and provide a foundation for selecting optimal computational parameters.

## 2 Methods

### 2.1 Theory of NEO‐DFT

We summarize the main aspects of the theory behind NEO‐DFT. For a more extensive representation we refer the reader to the publications by the Hammes‐Schiffer group who developed the method [26, 27, 34, 35].

In NEO‐DFT, selected nuclei—here protons—are treated as quantum particles like the electrons [26]. The total energy of a system in the NEO‐DFT framework can be written as a functional of protonic (ρ^p^) and electronic densities (ρ^e^) (Equation (1)).

(1) $$\begin{array}{c} E\left\lfloor \rho^{e},\rho^{p}\right\rfloor =\left(T_{s}\left[\rho^{e}\right]+T_{s}\left[\rho^{p}\right]\right)+\left(V_{\mathrm{ext}}^{e}\left[\rho^{e}\right]+V_{\mathrm{ext}}^{p}\left[\rho^{p}\right]\right)\\ +\left(J_{\mathrm{ee}}\left[\rho^{e}\right]+J_{\mathrm{pp}}\left[\rho^{p}\right]+J_{\mathrm{ep}}\left[\rho^{e},\rho^{p}\right]\right)\\ +\left(E_{\mathrm{xc}}^{e}\left[\rho^{e}\right]+E_{\mathrm{xc}}^{p}\left[\rho^{p}\right]+E^{\mathrm{epc}}\left[\rho^{e},\rho^{p}\right]\right) \end{array}$$

The terms in Equation (1) represent (in the order given in the equation): the noninteracting kinetic energy of the electrons and protons; the interaction of the external potential with the electrons and protons; the electron–electron, proton–proton, and electron–proton Coulombic interactions; the exchange‐correlation energies of the electrons and protons; and the electron–proton correlation energy. Within the NEO‐DFT framework, the existing exchange correlation functionals for electronic interactions can be used. In addition to the electron–proton interaction, they describe the kinetic energy differences and single‐component exchange‐correlation energies [36]. Due to the spatial localization of the quantum proton, the proton–proton exchange and correlation are negligible [27, 37]. However, proper treatment of electron–proton correlation is required for getting good protonic densities and energies. So far, a few electron–proton correlation (EPC) functionals have been designed [37, 38] such as epc17 [39] and epc19 [27]. The epc17 is of the local density approximation (LDA) type, and its mathematical form is given by,

(2) $$E_{\mathrm{epc}}\left[\rho^{e},\rho^{p}\right]=-\int \frac{\rho^{e}\left(r\right)\rho^{p}\left(r\right)}{a-b{\left[\rho^{e}\left(r\right)\rho^{p}\left(r\right)\right]}^{\frac{1}{2}}+c\rho^{e}\left(r\right)\rho^{p}\left(r\right)}\;dr$$

Where a, b, and c are parameters. Their values were determined by fitting to the proton density of the FHF^−^ molecule. This EPC is known as epc17‐1 with a, b, and c values determined to be 2.35, 2.4, and 3.2 respectively. Epc17‐1 has been proven to produce more accurate proton densities than previously reported approaches for correlating electronic and protonic densities [40, 41]. Since our study focuses on energies, we used the modified version of this EPC known as epc17‐2 [29]. The parameters set of epc17‐2 were determined by fitting to zero‐point energies of FHF^−^ and HCN as obtained from grid‐based calculations, resulting in a, b, and c values of 2.35, 2.4, and 6.6 respectively.

The functional epc‐19 is a generalized gradient approximation (GGA) type EPC that not only depends on the electronic and protonic densities but also on their gradient [27].

$$E_{\mathrm{epc}}\left[\rho^{e},\rho^{p},\nabla \rho^{e},\nabla \rho^{p}\right]=-\int \frac{\rho^{e}\left(r\right)\rho^{p}\left(r\right)}{a-b{\left[\rho^{e}\left(r\right)\rho^{p}\left(r\right)\right]}^{\frac{1}{2}}+c\rho^{e}\left(r\right)\rho^{p}\left(r\right)}$$

(3) $$\begin{array}{c} \left\{1-d\left(\frac{{\left[\rho^{e}\left(r\right)\rho^{p}\left(r\right)\right]}^{-\frac{1}{3}}}{{\left(1+m_{p}\right)}^{2}}\times \left[m_{p}^{2}\frac{\nabla^{2}\rho^{e}\left(r\right)}{\rho^{e}\left(r\right)}-2m_{p}\frac{\nabla \rho^{e}\left(r\right).\nabla \rho^{p}\left(r\right)}{\rho^{e}\left(r\right)\rho^{p}\left(r\right)}+\frac{\nabla^{2}\rho^{p}\left(r\right)}{\rho^{p}\left(r\right)}\right]\right)\right.\\ \left.\exp\left[\frac{-k}{{\left[\rho^{e}\left(r\right)\rho^{p}\left(r\right)\right]}^{\frac{1}{6}}}\right]\right\}\;dr \end{array}$$

The epc‐19 has additional parameters d and k and depends on the gradient as well as the mass of the proton (m_p_).

### 2.2 Computational Details

All calculations were performed with Q‐Chem 6.0 [42]. We used the DIIS (Direct Inversion in the Iterative Subspace) [43, 44] algorithm and SCF (self‐consistent field) convergence criteria of 10^−5^ Hartree. The precision of various NEO‐DFT parameters was probed across a set of 72 molecules (see Supporting Information for a list). We decided to apply the typical strategy in benchmark studies to vary one component at a time while keeping the others constant. The proton affinity of compound A is the negative of the enthalpy change associated with the protonation reaction.

$$A+H^{+}\to AH^{+}$$

Where the enthalpy of the reaction (ΔH) is given as:

(4) PA=−ΔH=−ΔE+RT

Where ΔE is the reaction energy. The energy term can be approximated as the overall sum of four energy terms (electronic, translational, rotational and vibrational) as below:

(5) $$\mathrm{\Delta E}\left(T\right)=\Delta E_{\text{elec}}+\Delta E_{\text{trans}}\left(T\right)+\Delta E_{\mathrm{rot}}\left(T\right)+\Delta E_{\mathrm{vib}}\left(T\right)$$

The change in translational energy upon protonation is −3/2 RT. Since the incoming proton has no rotational kinetic energy, the change in rotational energy is assumed to be negligible. So, the proton affinity can be represented as

(6) $$\mathrm{PA}\left(A\right)=\Delta E_{\text{elec}}+\Delta E_{\mathrm{vib}}+\frac{5}{2}\mathrm{RT}$$

NEO does include vibrational energy of the NEO center (incoming quantum proton) to the total energy calculation, and by considering that vibrational energies of the classical nuclei stay unchanged upon protonation, this approximation has been proven to be reliable in a previous study [45]. The PA can be written as:

(7) $$\mathrm{PA}\left(A\right)=E_{A}-E_{\mathrm{AH}+}+\frac{5}{2}\mathrm{RT}$$

Where E_AH+_ and E_A_ are the energies of species AH^+^ and A computed with NEO‐DFT and DFT, respectively. The enthalpy of the proton H^+^—also in the case of treating it as a quantum particle—is given solely as translational energy.

Eleven electronic density functionals were tested: B3LYP [46], PBE0 [47], TPSSh [48], X3LYP [49], B97‐D [50], CAM‐B3LYP [51], M06 [52], M11 [53], MN12‐SX [54], BP86, and PW91 [55]. The functionals were chosen from various classes comprising BP86, PW91, and B97‐D from GGA (Generalized Gradient Approximation), TPSSh and M06 from global hybrid meta‐GGA (meta‐Generalized Gradient Approximation), B3LYP, PBE0, and X3LYP from global hybrid functionals, CAM‐B3LYP from range‐separated functionals, and MN12‐SX and M11 from range‐separated hybrid meta‐GGA. The choice of functionals was based on the goal to achieve a broad spectrum of exchange‐correlation functional approximations as well as a strong use in computational chemistry literature. To investigate the importance of electron‐proton correlation, NEO‐DFT calculations without epc (No‐epc), with epc17‐2, and epc19 were carried out [27, 39]. Similarly, the size of the nuclear basis set was gradually increased from 2s2p2d to 10s10p10d with exponents ranging from 4 to 512, employing a bit larger exponents to mitigate convergence issues typically encountered in NEO calculations with too diffuse nuclear basis sets having small exponents [27, 28]. Apart from these three parameters, electronic basis sets, namely def2‐SVP, def2‐TZVP, and def2‐QZVP, were evaluated. We pre‐optimized all structures with BP86/def2‐TZVP to provide a good starting point for the (computationally demanding) NEO‐DFT optimizations. Then, all structures were fully optimized at the NEO‐DFT level stated in the manuscript. In all NEO‐DFT calculations, the added proton in AH^+^ was treated quantum mechanically, while all other atoms were treated classically.

## 3 Results

### 3.1 Functionals

**FIGURE 1:**
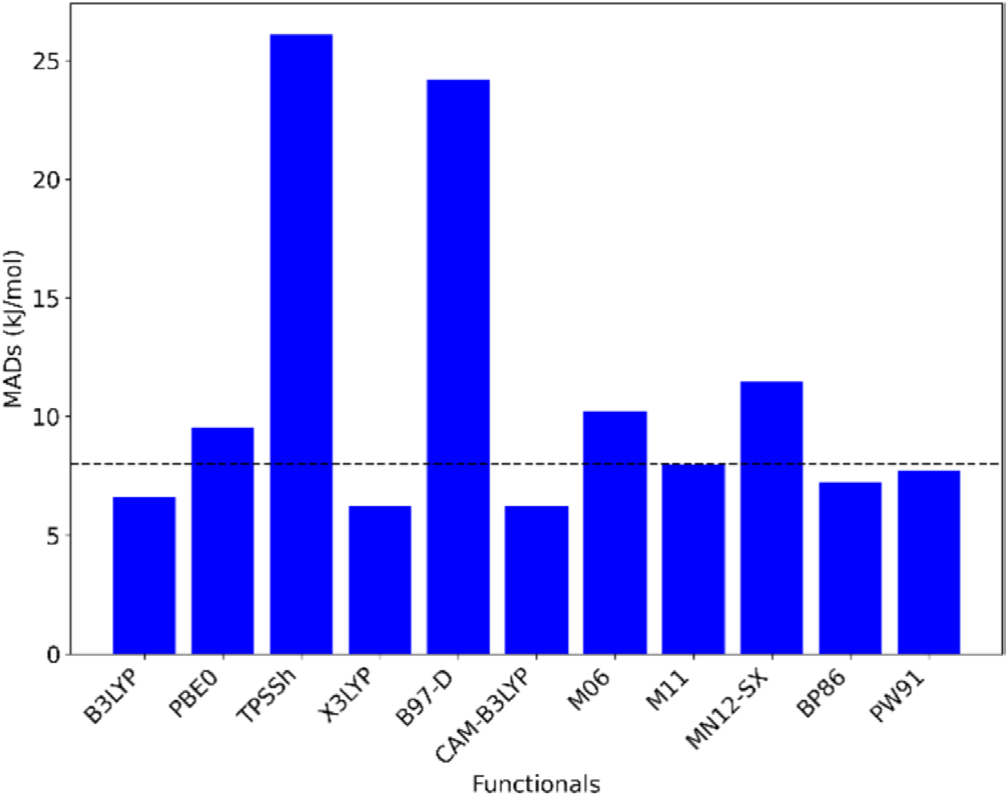
Comparison of MADs for different electronic density functionals w.r.t. experimental data [30] together with a dashed line representing the experimental uncertainty value.

We selected a range of 11 well‐known functionals to assess their respective accuracies and performance in our study. The other computational parameters were chosen to be def2‐TZVP as the electronic basis set, epc17‐2 as the electron‐proton exchange‐correlation functional and 2s2p2d as the nuclear basis set. The mean absolute deviation (MAD) of the computed PAs with respect to experimental data [56] is shown in Figure 1 (data are found in Table S2 of the Supporting Information).

Among the 11 selected functionals, the MAD values of six functionals (PW91, BP86, M11, CAM‐B3LYP, X3LYP, and B3LYP) were found to be lower than the corresponding experimental uncertainty value (8 kJ/mol). In the assessment of all considered functionals, TPSSh exhibited the largest MAD value (26.1 kJ/mol). Conversely, CAM‐B3LYP demonstrated the best performance among the tested functionals, showing the lowest mean absolute deviation value of 6.2 kJ/mol, however just by a small margin compared to, for example, B3LYP and X3LYP. In the comparison across different classes of exchange‐correlation (XC) functionals, the average MAD values were found to be 13.0 kJ/mol for GGA, 9.8 kJ/mol for meta GGA, 7.4 kJ/mol for global hybrids, 6.3 kJ/mol for range‐separated hybrids (RSH), and 9.8 kJ/mol for meta‐GGA RSHs. These results show that RSH and global hybrid XCs provide the most accurate PAs.

**FIGURE 2:**
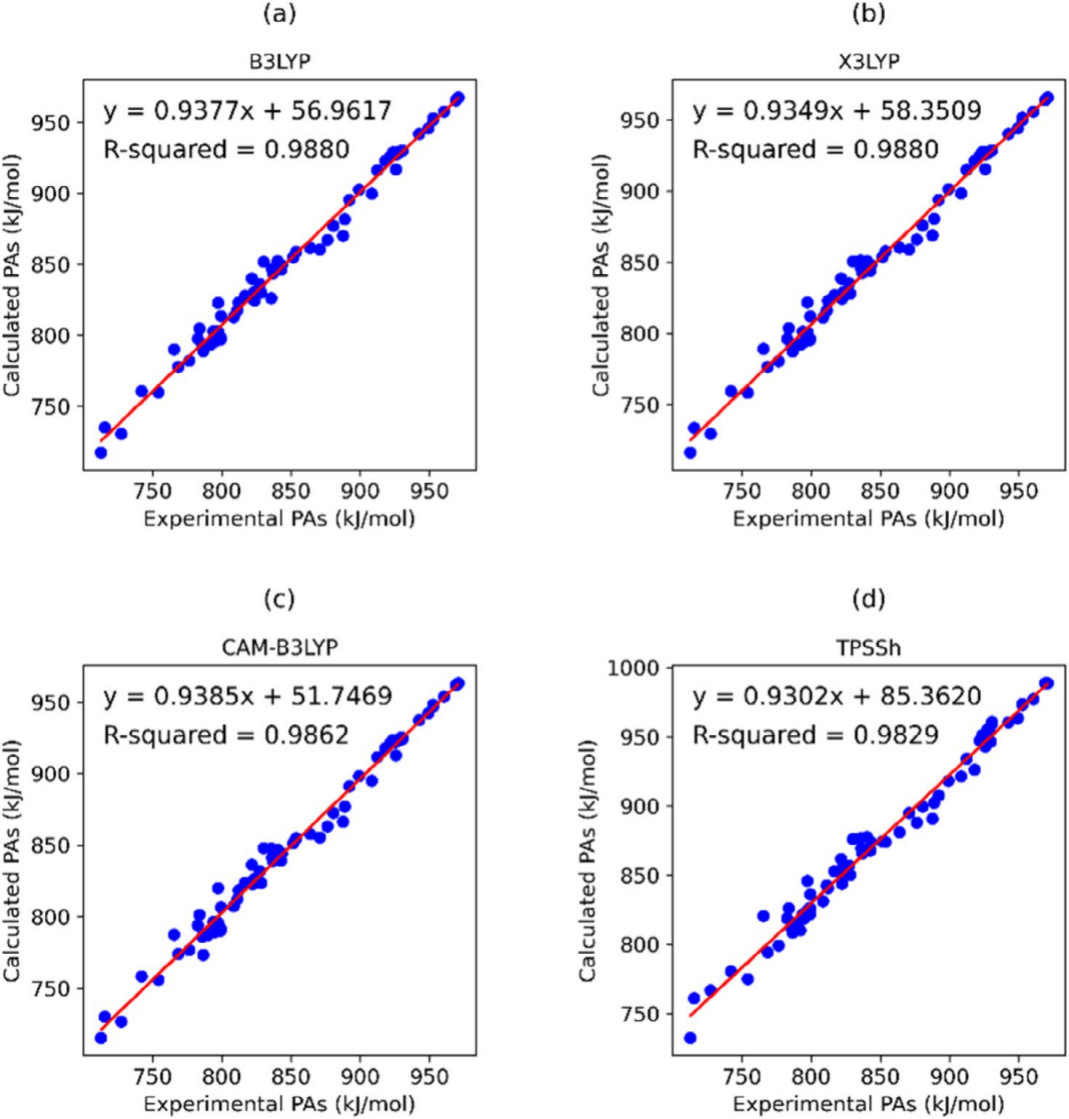
Correlation between experimental [30] and calculated PAs from 3 best (B3LYP, X3LYP, CAM‐B3LYP) and the worst functional (TPSSh) together with linear regression (red line).

Figure S1 depicts the relationship between the experimental PA values and the corresponding predicted PA values obtained from different XC functionals, indicating the precision of PA prediction. The calculated R^2^ values range from 0.823 to 0.988. Notably, three functionals B3LYP, X3LYP, and M11 demonstrated the closest alignment to experimental values, exhibiting an R^2^ value of 0.988. Conversely, the often‐used functional B97‐D yielded a weaker correlation, with an R^2^ value of 0.823. Figure 2 shows the relationship between the experimental values and predicted PA values obtained from 3 best (B3LYP, X3LYP, and CAM‐B3LYP) XC functionals and 1 of the worst (TPSSh) XC functionals in terms of MAD values. It is interesting to note that among all the functionals CAM‐B3LYP has the lowest MAD value, still it is slightly less precise than B3LYP and X3LYP in terms of R^2^ values.

### 3.2 Electron Proton Correlation Functionals

**FIGURE 3:**
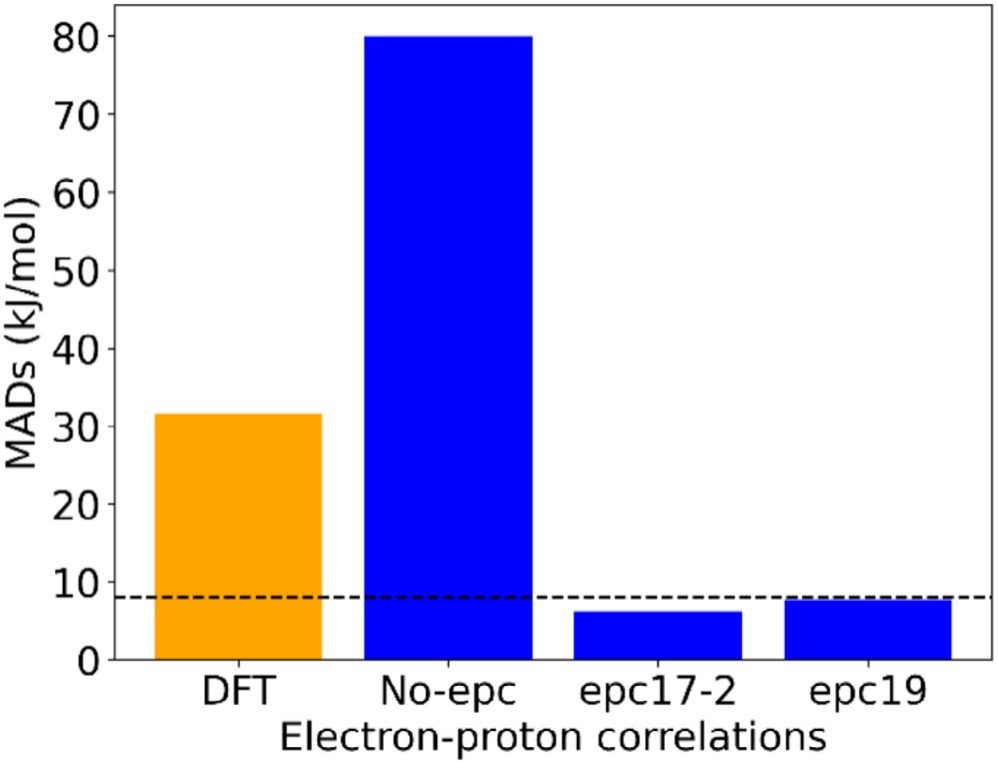
Comparison of MADs for different EPCs (in blue otherwise highlighted in different color) and (non‐NEO) DFT w.r.t. experimental data [30] together with a dashed line representing the experimental uncertainty value.

The proton affinities with various electron proton correlation functionals for the set of 72 molecules were computed using CAM‐B3LYP/*epc*/def2‐TZVP/2s2p2d (NEO‐DFT) and at CAM‐B3LYP/def2‐TZVP (DFT level) and compared with experimental proton affinity values. Figure 3 depicts the MAD values computed with different EPCs and DFT (without NEO) (data are found in Table S3 of the Supporting Information). Evident from the depicted figure, the lack of electron proton correlation (No‐epc) results in a notable mean absolute deviation value of 80.0 kJ/mol. The key reason for such elevated MAD values is the over‐localization of the proton densities generated when no electron proton correlation is used, offering evidence of the impact exerted by not capturing such correlation effects. The mean absolute deviation values for epc17‐2 and epc19 are 6.2 and 7.7 kJ/mol respectively. It is interesting to note here that epc17‐2 is an LDA type while epc19 is a GGA type functional but both approaches perform essentially equally well. However, it should be noted that the original version of epc17 (epc17‐1) was solely developed to provide reasonable protonic densities, but it was unable to provide good energies. So, the parameters were optimized again with zero‐point energies of FHF^−^ and HCN to obtain a new set of parameters (for epc17‐2) that can not only provide good protonic densities but also good energies. These results underscore the pivotal role of incorporating proper electron proton correlation effects in attaining accurate computational proton affinity values within NEO‐DFT. The results show that when EPC (epc17‐2 or epc19) is incorporated, NEO‐DFT provides accurate proton affinities, indicating that using proper electron proton correlation improves the accuracy. We also computed proton affinities for the given set of molecules on DFT (CAM‐B3LYP/def2‐TZVP) level without NEO (referred to as DFT in the following). As shown in Figure 3, DFT performs worse than NEO‐DFT when proper electron proton correlation is used in NEO‐DFT calculations (MAD = 31.6 kJ/mol). This is mainly due to the inclusion of the zero‐point energy of the quantum proton at the NEO‐DFT level, which is lacking in our DFT‐only calculations. Of course, ZPE correction can be achieved by invoking the harmonic approximation (DFT + ZPE). But NEO‐DFT directly accounts for these effects.

**FIGURE 4:**
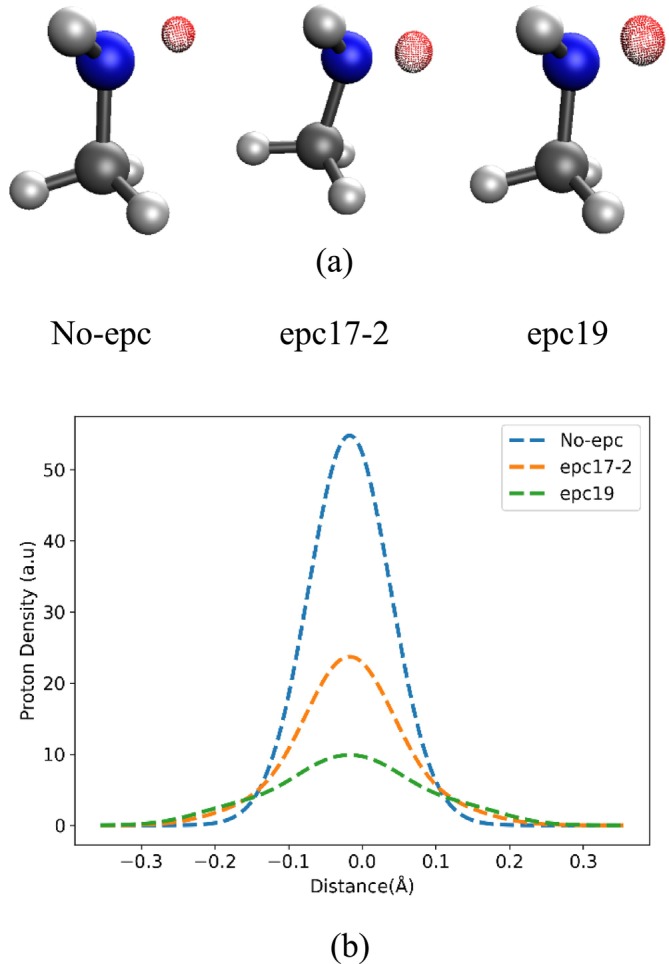
3D (a) and off‐axis 1D (b) slice of quantum proton density of CH_3_NH_2_(H^+^) calculated with different epcs at the NEO‐DFT level.

To visually capture the relationship between experimental and predicted values, scatter graphs were plotted for each EPC and DFT, as shown in Figure S2 (see Supporting Information). The graphs exhibit consistent trends across the three EPCs and DFT, showcasing a significant agreement between experimental and predicted PA values. The R^2^ values were found to be remarkably similar for all three epcs and DFT. The R^2^ values were 0.985, 0.986, 0.985, and 0.986 for No‐epc, epc17‐2, epc19 and DFT respectively. This shows that the incorporation of electron‐proton correlation effects improves the absolute agreement with experimental proton affinity data, but the trend is already correctly captured when treating the H atoms classically. Figure 4 shows the 3D and 1D proton densities of the incoming proton in one example molecule (CH_3_NH_2_(H^+^)). It can be clearly seen from the figure that No‐epc create overly localized proton densities, while epc17‐2 and epc19 produce quite delocalized protonic densities.

### 3.3 Electronic Basis Sets

**FIGURE 5:**
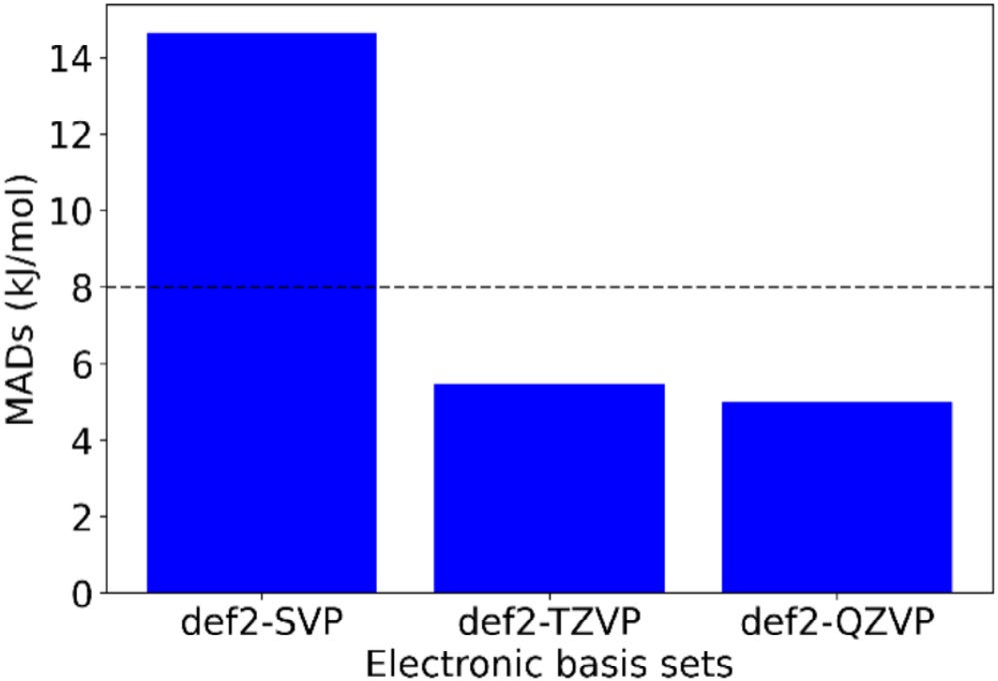
Comparison of MADs for different electronic basis sets w.r.t. experimental data [30] together with dash line representing the experimental uncertainty value.

As a next step, we checked the influence of varying the basis set for the electrons in the system (EBSs: electronic basis sets). We wanted to see if the usual experience of quantum chemistry regarding basis set convergence are still true when treating nuclei as quantum particles. Proton affinities were thus calculated for a set of 72 molecules at the CAM‐B3LYP/epc17‐2/*EBSs*/2s2p2d level (and the mean absolute deviation values) are represented in Figure 5 (data are found in Table S4 of the Supporting Information). Calculations for def2‐SVP and def2‐TZVP were completed for all (72) molecules but for def2‐QZVP basis set only a set of 42 molecules (see Supporting Information) was successfully completed due to convergence issues. Figure 5 shows a comparison of MAD values for those 42 molecules with experimental data. With the increasing size of the EBS, the mean absolute deviation values for def2‐SVP, def2‐TZVP, and def2‐QZVP converge at 14.6, 5.5, and 5.0 kJ/mol, respectively, thus confirming basis set convergence trends observed earlier for classical nuclei. Indeed, in comparison to the MAD values of def2‐QZVP (5.0 kJ/mol) and def2‐TZVP (5.5 kJ/mol), the mean absolute deviation value of def2‐SVP (14.6 kJ/mol) stands large, signifying a disparity in predictive accuracy among the three basis sets. Moreover, this disparity is further accentuated by the fact that the MAD value for def2‐SVP exceeds the typical range of experimental uncertainty value, indicating the importance of employing larger basis sets like def2‐TZVP and def2‐QZVP to achieve more precise results. It is noteworthy that while the MAD value of def2‐SVP (14.6 kJ/mol) may be relatively larger the proximity of the other two MAD values (5.5 kJ/mol for def2‐TZVP and 5.0 kJ/mol for def2‐QZVP) to both the experimental uncertainty value (8 kJ/mol) and the chemical accuracy threshold (4.82 kJ/mol) speaks about the precision and reliability of those basis sets. Since def2‐QZVP is computationally much more demanding, def2‐TZVP is the most efficient basis set for NEO‐DFT computations, in line with the findings for classical nuclei.

For each electronic basis set, a scatter plot was generated (Figure S3), visualizing the relationship between experimental PA values and the corresponding predicted PA values. The quantitative assessment of predictive accuracy was carried out using the R^2^ values, which gauge the extent of agreement between experimental and predicted data. The def2‐SVP basis set yielded an R^2^ value of 0.985, while both the def2‐TZVP and def2‐QZVP basis sets showed identical R^2^ values of 0.986.

### 3.4 Nuclear Basis Sets

**FIGURE 6:**
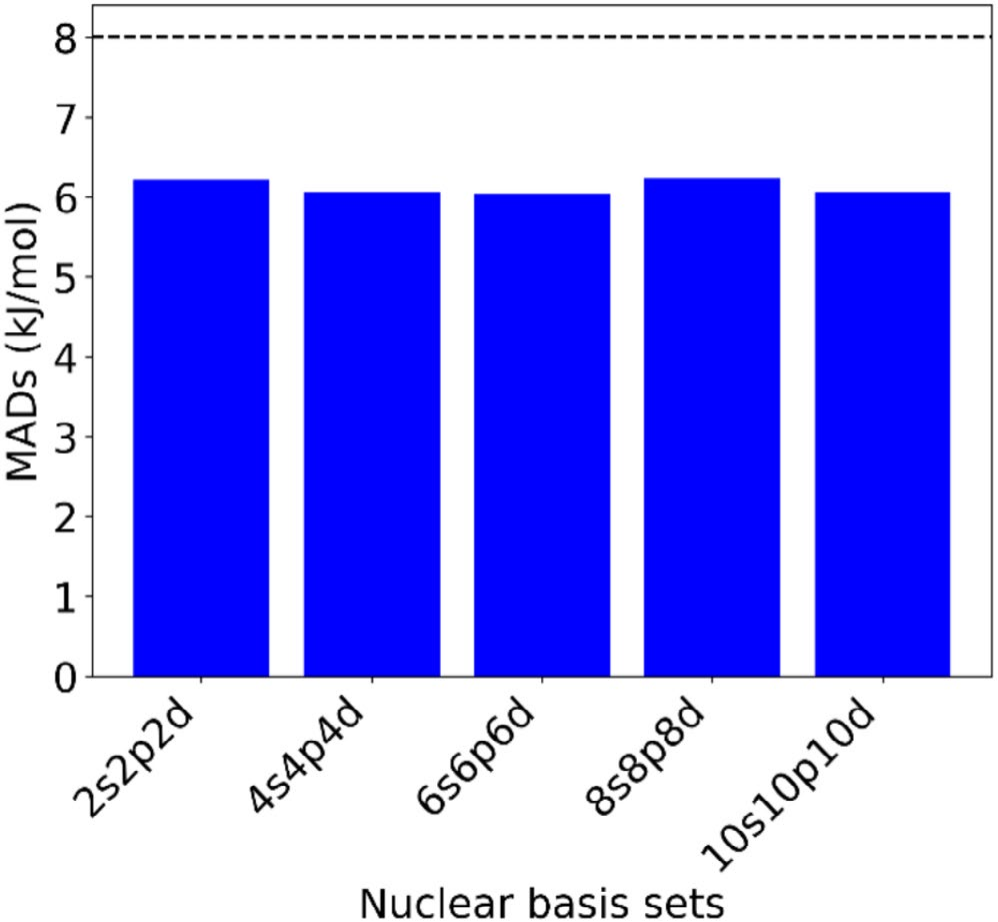
Comparison of MADs for different nuclear basis sets w.r.t. experimental data [30] together with dash line representing experimental uncertainty value.

In a NEO‐DFT computation, not only the basis set for the electrons but also for the quantum nuclei must be chosen. Thus, the proton affinity values for a set of 72 molecules were calculated at the CAM‐B3LYP/epc17‐2/def2‐TZVP/*NBSs* level (where NBSs stands for nuclear basis sets) by gradually increasing the size of the NBS from 2s2p2d to 10s10p10d. Figure 6 demonstrates that all NBSs exhibit very low mean absolute deviation values, indicating a high level of agreement between the predicted and experimental data (computational data are found in Table S5 of the Supporting Information). The small variation observed in the MAD values across different NBSs suggests that increasing nuclear basis set size has a minimal impact on the computed proton affinity values. The MAD values fall well within the range of the experimental uncertainty value. The MAD values resulting from different NBSs only vary at the decimal point level (6.05–6.24 kJ/mol) which renders them essentially equal. It can thus be concluded that even the smallest NBSs like 2s2p2d or 4s4p4d are sufficient for the computation of PAs. This result is consistent with previous benchmarking on another series of NBSs (PB4, PB5, and PB6) where comparatively smaller nuclear basis sets such as PB4‐D were able to accurately predict the ground state properties [28].

For each NBS, we constructed scatter plots as shown in Figure S4 (see Supporting Information), visually depicting the correspondence between experimental PA values and the computed PA values. Across all five nuclear basis sets, the scatter plots consistently revealed a tight clustering of data points along the diagonal line, indicating a strong alignment between experimental and predicted PA values. All five basis sets yielded identical R^2^ values of 0.986. This uniformity in R^2^ values demonstrates a consistent correlation between experimental and predicted PA values, regardless of the specific nuclear basis set employed.

## 4 Conclusion

Through a systematic benchmarking process, we have examined various computational parameters of the NEO‐DFT (Nuclear‐Electronic Orbital Density Functional Theory) method, highlighting their influence on the accuracy of computations for the example of proton affinities of a set of 72 small organic molecules. The findings offer first insights into selecting optimal parameters for accuracy and reliability in NEO‐DFT computations. In the evaluation of various exchange‐correlation functionals, hybrid density functionals demonstrated the best performance for accurately predicting proton affinities. Among all the evaluated functionals, CAM‐B3LYP has the lowest MAD value of 6.2 kJ/mol. With an R^2^ value of 0.988, B3LYP and X3LYP stand out as the most precise functionals while B97‐D is less precise with an R^2^ value of 0.823. The MAD values of different EPCs emphasize the significance of incorporating proper electron‐proton correlation in the calculation of proton affinities, while the actual choice of the EPC had a minor impact with 6.2 and 7.7 kJ/mol for epc17‐2 and epc19 respectively. The comparison between NEO‐DFT and only DFT calculations shows that NEO‐DFT produces more accurate results than DFT when a proper EPC is used highlighting the importance of NQEs when calculating proton affinities. Similarly, our investigation reveals that the accuracy of proton affinities is significantly influenced by the choice of the electronic basis set similar to a typical DFT computation, with def2‐QZVP having the lowest MAD value of 5.0 kJ/mol. A basis set of triple‐zeta quality (here: def2‐TZVP) is sufficient, while double‐zeta quality (def2‐SVP) only gives qualitative agreement with experiment. Unlike electronic basis sets, nuclear basis sets show a limited influence and no apparent systematic trend, underlining their minor contribution to the overall precision of proton affinity calculations. Our results show that NEO‐DFT provides an alternative approach to predict proton affinity values by considering the influence of nuclear quantum effects on the electronic structure.

## Supporting information


Data S1.

## Data Availability

All data underlying these results can be found in the openly accessible database Zenodo: 10.5281/zenodo.11580957.

## References

[1] S. Habershon , T. E. Markland , and D. E. Manolopoulos , “Competing Quantum Effects in the Dynamics of a Flexible Water Model,” Journal of Chemical Physics 131 (2009): 2. 10.1063/1.3167790 19603998

[2] X.‐Z. Li , B. Walker , and A. Michaelides , “Quantum Nature of the Hydrogen Bond,” Proceedings of the National Academy of Sciences 108, no. 16 (2011): 6369–6373.

[3] M. Ceriotti , J. Cuny , M. Parrinello , and D. E. Manolopoulos , “Nuclear Quantum Effects and Hydrogen Bond Fluctuations in Water,” Proceedings of the National Academy of Sciences 110, no. 39 (2013): 15591–15596. 10.1073/pnas.1308560110 PMC3785726 24014589

[4] D. Marx , M. E. Tuckerman , J. Hutter , and M. Parrinello , “The Nature of the Hydrated Excess Proton in Water,” Nature 397, no. 6720 (1999): 601–604.

[5] J. A. Morrone and R. Car , “Nuclear Quantum Effects in Water,” Physical Review Letters 101, no. 1 (2008): 017801. 18764152 10.1103/PhysRevLett.101.017801

[6] G. S. Fanourgakis , G. K. Schenter , and S. S. Xantheas , “A Quantitative Account of Quantum Effects in Liquid Water,” Journal of Chemical Physics 125 (2006): 14. 10.1063/1.2358137 17042571

[7] L. Masgrau , A. Roujeinikova , L. O. Johannissen , et al., “Atomic Description of an Enzyme Reaction Dominated by Proton Tunneling,” Science 312, no. 5771 (2006): 237–241. 16614214 10.1126/science.1126002

[8] D. G. Truhlar , “Tunneling in Enzymatic and Nonenzymatic Hydrogen Transfer Reactions,” Journal of Physical Organic Chemistry 23, no. 7 (2010): 660–676.

[9] T. Pirali , M. Serafini , S. Cargnin , and A. A. Genazzani , “Applications of Deuterium in Medicinal Chemistry,” Journal of Medicinal Chemistry 62, no. 11 (2019): 5276–5297. 30640460 10.1021/acs.jmedchem.8b01808

[10] E. M. Russak and E. M. Bednarczyk , “Impact of Deuterium Substitution on the Pharmacokinetics of Pharmaceuticals,” Annals of Pharmacotherapy 53, no. 2 (2019): 211–216. 30136594 10.1177/1060028018797110

[11] R. M. C. di Martino , B. D. Maxwell , and T. Pirali , “Deuterium in Drug Discovery: Progress, Opportunities and Challenges,” Nature Reviews Drug Discovery 22 (2023): 1–23. 10.1038/s41573-023-00703-8 PMC10241557 37277503

[12] X. Yang , H. Ben , and A. J. Ragauskas , “Recent Advances in the Synthesis of Deuterium‐Labeled Compounds,” Asian Journal of Organic Chemistry 10, no. 10 (2021): 2473–2485.

[13] M. Shao , J. Keum , J. Chen , et al., “The Isotopic Effects of Deuteration on Optoelectronic Properties of Conducting Polymers,” Nature Communications 5, no. 1 (2014): 3180, 10.1038/ncomms4180. 24458188

[14] C. Shi , X. Zhang , C.‐H. Yu , Y.‐F. Yao , and W. Zhang , “Geometric Isotope Effect of Deuteration in a Hydrogen‐Bonded Host–Guest Crystal,” Nature Communications 9, no. 1 (2018): 481. 10.1038/s41467-018-02931-8 PMC5797174 29396512

[15] Y. Ikabata , Y. Imamura , and H. Nakai , “Interpretation of Intermolecular Geometric Isotope Effect in Hydrogen Bonds: Nuclear Orbital Plus Molecular Orbital Study,” Journal of Physical Chemistry A 115, no. 8 (2011): 1433–1439. 21306139 10.1021/jp111062n

[16] M. Shibl , M. Tachikawa , and O. Kühn , “The Geometric (H/D) Isotope Effect in Porphycene: Grid‐Based Born–Oppenheimer Vibrational Wavefunctions vs. Multi‐Component Molecular Orbital Theory,” Physical Chemistry Chemical Physics 7, no. 7 (2005): 1368–1373. 19787956 10.1039/b500620a

[17] M. Ceriotti , W. Fang , P. G. Kusalik , et al., “Nuclear Quantum Effects in Water and Aqueous Systems: Experiment, Theory, and Current Challenges,” Chemical Reviews 116, no. 13 (2016): 7529–7550. 27049513 10.1021/acs.chemrev.5b00674

[18] T. E. Markland and M. Ceriotti , “Nuclear Quantum Effects Enter the Mainstream,” Nature Reviews Chemistry 2, no. 3 (2018): 109.

[19] W. Fang , J. Chen , M. Rossi , Y. Feng , X.‐Z. Li , and A. Michaelides , “Inverse Temperature Dependence of Nuclear Quantum Effects in DNA Base Pairs,” Journal of Physical Chemistry Letters 7, no. 11 (2016): 2125–2131. 27195654 10.1021/acs.jpclett.6b00777 PMC4933496

[20] M. Rossi , W. Fang , and A. Michaelides , “Stability of Complex Biomolecular Structures: Van der Waals, Hydrogen Bond Cooperativity, and Nuclear Quantum Effects,” Journal of Physical Chemistry Letters 6, no. 21 (2015): 4233–4238. 26722963 10.1021/acs.jpclett.5b01899

[21] C. Zhang and A. Michaelides , “Quantum Nuclear Effects on the Location of Hydrogen Above and Below the Palladium (100) Surface,” Surface Science 605, no. 7–8 (2011): 689–694.

[22] T. Udagawa , T. Tsuneda , and M. Tachikawa , “Electron‐Nucleus Correlation Functional for Multicomponent Density‐Functional Theory,” Physical Review A 89, no. 5 (2014): 052519.

[23] Y. Shigeta , H. Takahashi , S. Yamanaka , M. Mitani , H. Nagao , and K. Yamaguchi , “Density Functional Theory Without the Born‐Oppenheimer Approximation and Its Application,” International Journal of Quantum Chemistry 70, no. 4–5 (1998): 659–669.

[24] H. Nakai , “Nuclear Orbital Plus Molecular Orbital Theory: Simultaneous Determination of Nuclear and Electronic Wave Functions Without Born–Oppenheimer Approximation,” International Journal of Quantum Chemistry 107, no. 14 (2007): 2849–2869.

[25] L. Hasecke and R. A. Mata , “Nuclear Quantum Effects Made Accessible: Local Density Fitting in Multicomponent Methods,” Journal of Chemical Theory and Computation 19, no. 22 (2023): 8223–8233. 37920900 10.1021/acs.jctc.3c01055 PMC10687858

[26] F. Pavošević , T. Culpitt , and S. Hammes‐Schiffer , “Multicomponent Quantum Chemistry: Integrating Electronic and Nuclear Quantum Effects via the Nuclear–Electronic Orbital Method,” Chemical Reviews 120, no. 9 (2020): 4222–4253. 32283015 10.1021/acs.chemrev.9b00798

[27] Z. Tao , Y. Yang , and S. Hammes‐Schiffer , “Multicomponent Density Functional Theory: Including the Density Gradient in the Electron‐Proton Correlation Functional for Hydrogen and Deuterium,” Journal of Chemical Physics 151 (2019): 12. 10.1063/1.5119124 31575164

[28] Q. Yu , F. Pavošević , and S. Hammes‐Schiffer , “Development of Nuclear Basis Sets for Multicomponent Quantum Chemistry Methods,” Journal of Chemical Physics 152 (2020): 24. 10.1063/5.0009233 32610964

[29] K. R. Brorsen , Y. Yang , and S. Hammes‐Schiffer , “Multicomponent Density Functional Theory: Impact of Nuclear Quantum Effects on Proton Affinities and Geometries,” Journal of Physical Chemistry Letters 8, no. 15 (2017): 3488–3493. 28686449 10.1021/acs.jpclett.7b01442

[30] E. P. Hunter and S. G. Lias , “Evaluated Gas Phase Basicities and Proton Affinities of Molecules: An Update,” Journal of Physical and Chemical Reference Data 27, no. 3 (1998): 413–656, 10.1063/1.556018.

[31] J. W. Knight , X. Wang , L. Gallandi , et al., “Accurate Ionization Potentials and Electron Affinities of Acceptor Molecules III: A Benchmark ofGWMethods,” Journal of Chemical Theory and Computation 12, no. 2 (2016): 615–626. 26731609 10.1021/acs.jctc.5b00871

[32] S. Shil , D. Bhattacharya , S. Sarkar , and A. Misra , “Performance of the Widely Used Minnesota Density Functionals for the Prediction of Heat of Formations, Ionization Potentials of Some Benchmarked First Row Transition Metal Complexes,” Journal of Physical Chemistry A 117, no. 23 (2013): 4945–4955. 23701489 10.1021/jp400397r

[33] S. McKechnie , G. H. Booth , A. J. Cohen , and J. M. Cole , “On the Accuracy of Density Functional Theory and Wave Function Methods for Calculating Vertical Ionization Energies,” Journal of Chemical Physics 142 (2015): 19. 10.1063/1.4921037 26001454

[34] A. Chakraborty , M. V. Pak , and S. Hammes‐Schiffer , “Development of Electron‐Proton Density Functionals for Multicomponent Density Functional Theory,” Physical Review Letters 101, no. 15 (2008): 153001. 18999594 10.1103/PhysRevLett.101.153001

[35] M. V. Pak , A. Chakraborty , and S. Hammes‐Schiffer , “Density Functional Theory Treatment of Electron Correlation in the Nuclear−Electronic Orbital Approach,” Journal of Physical Chemistry A 111, no. 20 (2007): 4522–4526. 17441701 10.1021/jp0704463

[36] N. Gidopoulos , “Kohn‐Sham Equations for Multicomponent Systems: The Exchange and Correlation Energy Functional,” Physical Review B 57, no. 4 (1998): 2146–2152.

[37] K. R. Brorsen , P. E. Schneider , and S. Hammes‐Schiffer , “Alternative Forms and Transferability of Electron‐Proton Correlation Functionals in Nuclear‐Electronic Orbital Density Functional Theory,” Journal of Chemical Physics 149 (2018): 4. 10.1063/1.5037945 30068159

[38] A. Sirjoosingh , M. V. Pak , and S. Hammes‐Schiffer , “Multicomponent Density Functional Theory Study of the Interplay Between Electron‐Electron and Electron‐Proton Correlation,” Journal of Chemical Physics 136, no. 17 (2012): 174114. 22583217 10.1063/1.4709609

[39] Y. Yang , K. R. Brorsen , T. Culpitt , M. V. Pak , and S. Hammes‐Schiffer , “Development of a Practical Multicomponent Density Functional for Electron‐Proton Correlation to Produce Accurate Proton Densities,” Journal of Chemical Physics 147, no. 11 (2017): 114113. 28938833 10.1063/1.4996038

[40] A. Sirjoosingh , M. V. Pak , K. R. Brorsen , and S. Hammes‐Schiffer , “Quantum Treatment of Protons With the Reduced Explicitly Correlated Hartree‐Fock Approach,” Journal of Chemical Physics 142 (2015): 21. 10.1063/1.4921303 26049479

[41] T. Culpitt , K. R. Brorsen , M. V. Pak , and S. Hammes‐Schiffer , “Multicomponent Density Functional Theory Embedding Formulation,” Journal of Chemical Physics 145 (2016): 4. 10.1063/1.4958952 27475347

[42] E. Epifanovsky , A. T. Gilbert , X. Feng , et al., “Software for the Frontiers of Quantum CHEMISTRY: An overview of Developments in the Q‐Chem 5 Package,” Journal of Chemical Physics 155, no. 8 (2021): 084801‐1–084801‐59. 34470363 10.1063/5.0055522 PMC9984241

[43] P. Pulay , “Convergence Acceleration of Iterative Sequences. The Case of Scf Iteration,” Chemical Physics Letters 73, no. 2 (1980): 393–398.

[44] P. Pulay , “ImprovedSCFconvergence Acceleration,” Journal of Computational Chemistry 3, no. 4 (1982): 556–560.

[45] F. Pavošević , T. Culpitt , and S. Hammes‐Schiffer , “Multicomponent Coupled Cluster Singles and Doubles Theory Within the Nuclear‐Electronic Orbital Framework,” Journal of Chemical Theory and Computation 15, no. 1 (2018): 338–347, 10.1021/acs.jctc.8b01120. 30525610

[46] A. D. Becke , “Density‐Functional Thermochemistry. I. The Effect of the Exchange‐Only Gradient Correction,” Journal of Chemical Physics 96, no. 3 (1992): 2155–2160.

[47] C. Adamo and V. Barone , “Toward Reliable Density Functional Methods Without Adjustable Parameters: The PBE0 Model,” Journal of Chemical Physics 110, no. 13 (1999): 6158–6170.

[48] V. Staroverov , G. Scuseria , J. Tao , and J. Perdew , “Comparative Assessment of a New Nonempirical Density Functional: Molecules and Hydrogen‐Bonded Complexes,” Journal of Chemical Physics 119 (2003): 12129–12137. 10.1063/1.4971853 28010100

[49] X. Xu and W. A. Goddard, III , “The X3LYP Extended Density Functional for Accurate Descriptions of Nonbond Interactions, Spin States, and Thermochemical Properties,” Proceedings of the National Academy of Sciences 101, no. 9 (2004): 2673–2677. 10.1073/pnas.0308730100 PMC374194 14981235

[50] S. Grimme , “Semiempirical GGA‐Type Density Functional Constructed With a Long‐Range Dispersion Correction,” Journal of Computational Chemistry 27, no. 15 (2006): 1787–1799. 16955487 10.1002/jcc.20495

[51] T. Yanai , D. P. Tew , and N. C. Handy , “A New Hybrid Exchange–Correlation Functional Using the Coulomb‐Attenuating Method (CAM‐B3LYP),” Chemical Physics Letters 393, no. 1–3 (2004): 51–57.

[52] Y. Zhao and D. G. Truhlar , “The M06 Suite of Density Functionals for Main Group Thermochemistry, Thermochemical Kinetics, Noncovalent Interactions, Excited States, and Transition Elements: Two New Functionals and Systematic Testing of Four M06‐Class Functionals and 12 Other Functionals,” Theoretical Chemistry Accounts 120 (2008): 215–241.

[53] R. Peverati and D. G. Truhlar , “Improving the Accuracy of Hybrid Meta‐GGA Density Functionals by Range Separation,” Journal of Physical Chemistry Letters 2, no. 21 (2011): 2810–2817.

[54] R. Peverati and D. G. Truhlar , “Screened‐Exchange Density Functionals With Broad Accuracy for Chemistry and Solid‐State Physics,” Physical Chemistry Chemical Physics 14, no. 47 (2012): 16187–16191. 23132141 10.1039/c2cp42576a

[55] J. P. Perdew , J. A. Chevary , S. H. Vosko , et al., “Atoms, Molecules, Solids, and Surfaces: Applications of the Generalized Gradient Approximation for Exchange and Correlation,” Physical Review B 46, no. 11 (1992): 6671–6687. 10.1103/physrevb.46.6671 10002368

[56] E. P. Hunter , and S. G. Lias , Journal of Physical and Chemical Reference Data 27 (1998): 413–656.

