# Supplementary material for: Optimizing Computational Parameters for Nuclear Electronic Orbital Density Functional Theory: A Benchmark Study on Proton Affinities
Source: J Comput Chem. 2025 Mar 18;46(8):e70082. doi: 10.1002/jcc.70082 (PMC11915487; doi:10.1002/jcc.70082)
Supplement: Supplementary file 1 — Data S1. [file JCC-46-0-s001.pdf]

Optimizing Computational Parameters for Nuclear Electronic Orbital Density Functional Theory: A Benchmark Study on Proton Affinities

Raza Ullah Khan, Ralf Tonner-Zech*

Table S1. Test set of 72 molecules and their experimental proton affinity (PA)[1] values (in kJ/mol).

Class of molecule	Serial No	Molecule	PAs(kJ/mol)
Amines	1	NH_3	853.60
	2	$CHF_2CH_2NH_2$	870.50
	3	$CH_2FCH_2NH_2$	892.00
	4	CH_3NH_2	899.00
	5	$CH_3CH_2NH_2$	912.00
	6	$CH_3(CH_2)_2NH_2$	917.80
	7	CH3(CH2)3NH2	921.50
	8	$CH_3(CH_2)_4NH_2$	923.50
	9	$(CH_3)_2CHNH_2$	923.80
	10	$(CH_3)_2CHCH_2NH_2$	924.80
	11	$CH_3(CH_2)_5NH_2$	927.50
	12	$CH_3(CH_2)_6NH_2$	927.00
	13	$CH_3(CH_2)_7NH_2$	928.90
	14	$CH_3(CH_2)_8NH_2$	930.00
	15	$CH_3(CH_2)_9NH_2$	930.52
Secondary amines	16	$(CH_3)_2NH$	929.50
	17	$CH_3CH_2NHCH_3$	942.20
	18	$(CH_3)_2CHNHCH_3$	952.30
	19	$(CH_3CH_2)_2NH$	952.40
Teriary amines	20	$(CH_3)_3N$	948.90
	21	$CH_3CH_2N(CH_3)_2$	960.23
	22	$(CH_3)_2CHCH_2N(CH_3)_2$	968.70
	23	$(CH_3)_2CHN(CH_3)_2$	970.60
Amides	24	$HCONH_2$	822.16
	25	$HCONHCH_3$	851.30
	26	CH_3CONH_2	863.60
	27	$CH_3CH_2CONH_2$	876.20
	28	$(CH_3)_2CHCONH_2$	880.31
	29	$(CH_3)_2NCHO$	887.50
	30	$CH_3CONHCH_2$	888.50

	31	$CH_3CON(CH_3)_2$	908.00
	32	$CH_3CON(C_2H_5)_2$	925.40
Esters	33	$HCOOCH_3$	782.50
	34	$HCO_2C_2H_5$	799.40
	35	$HCOOCH(CH_3)_2$	811.30
	36	$CH_3CO_2CH_3$	821.60
	37	$C_2H_5COOCH_3$	830.20
	38	$CH_3COOCH_2CH_3$	835.70
Alcohols	39	CH_2FCH_2OH	715.60
	40	CHF_2CH_2OH	727.40
	41	CH_3OH	754.30
	42	CH_3CH_2OH	776.40
	43	$CH_3(CH_2)_2OH$	786.50
	44	$CH_3(CH_2)_3OH$	789.20
	45	$(CH_3)_2CH_2OH$	793.70
	46	$CH_3(CH_2)_3OH$	789.20
	47	$CH_3(CH_2)_4OH$	795.00
	48	$CH_3(CH_2)_5OH$	799.00
	49	$CH_3(CH_2)_6OH$	799.00
	50	$CH_3(CH_2)_7OH$	799.00
Ethers	51	CH_3OCH_3	792.00
	52	$CH_3CH_2OCH_3$	808.60
	53	$(CH_3)_2CHOCH_3$	826.30
	54	$(C_2H_5)_2O$	828.40
	55	$(CH_3)_2CHOCH_2CH_3$	842.70
Aldehyde	56	CH_2O	712.90
	57	CH_3CHO	768.50
	58	CH_3CH_2CHO	786.00
	59	$(CH_3)_2CHCOH$	797.30
Ketones	60	$(CH_3)_2CO$	812.00
	61	$CH_3COC_2H_5$	827.30
	62	$(CH_3)_2CHCOCH_3$	836.30
	63	$(C_2H_5)_2CO$	836.80

	64	$(CH_3)_3COCH_3$	840.10
	65	$CH_3CH_2CH_2COCH_2CH_3$	843.20
Carboxylic acid	66	HCOOH	742.00
	67	CH_2FCOOH	765.40
	68	CH_3COOH	783.70
	69	C_2H_5COOH	797.20
	70	$(CH_3)_2CCOOH$	816.70
	71	$(CH_3)_2CCHCOOH$	823.00
	72	$CH_3(CH)_2COOH$	823.80

Table S2. Computed PAs (in kJ/mol) calculated at functional/Def2-TZVP/epc17-2/2s2p2d. where each functional is indicated in the table.

Serial No[1]	B3LYP	PBE0	TPSSh	X3LYP	B97-D	CAM-B3LYP	M06	M11	MN12-SX	BP86	PW91
1	859.02	866.58	874.02	857.68	869.84	854.90	859.92	860.60	864.32	859.44	859.05
2	860.36	866.63	895.11	858.90	870.40	855.49	877.23	867.01	880.90	861.08	860.23
3	895.43	901.00	907.45	894.04	896.18	891.34	901.56	901.85	904.78	894.33	893.43
4	902.70	907.90	918.29	901.25	907.03	898.30	896.90	905.79	899.90	900.92	900.03
5	916.46	909.65	934.11	914.90	912.50	911.61	910.36	920.18	913.38	915.00	914.21
6	922.84	928.21	926.12	921.28	906.37	917.86	920.49	923.44	921.02	921.66	920.93
7	926.54	916.37	947.35	924.99	898.69	921.45	925.38	926.72	925.24	925.40	924.72
8	928.97	934.30	951.27	927.42	889.59	923.81	929.00	928.68	928.23	927.99	927.33
9	927.05	932.25	946.07	925.38	908.96	921.81	923.51	928.07	924.19	925.79	925.02
10	927.18	932.56	948.50	925.56	893.43	921.98	924.11	927.61	924.94	926.18	939.94
11	928.60	933.41	952.95	926.98	879.97	923.11	921.42	933.41	925.00	927.15	926.60
12	929.36	934.14	955.12	927.73	869.20	923.80	922.09	934.60	925.73	927.96	927.42
13	929.78	934.50	957.13	928.16	858.26	924.18	922.78	935.33	926.31	928.30	927.81
14	930.05	934.79	958.95	928.43	847.14	924.47	923.22	936.11	926.74	928.59	927.86
15	930.10	935.01	960.70	928.71	834.50	924.67	923.65	936.70	927.10	928.43	927.87
16	929.73	933.16	946.32	928.13	922.89	925.57	919.13	935.54	921.63	926.03	924.52
17	942.03	945.61	960.30	940.34	925.77	937.53	931.05	948.16	933.20	938.80	937.37
18	951.85	955.54	973.83	950.13	905.14	947.18	945.94	953.91	945.10	948.85	947.52
19	953.16	956.84	973.36	951.41	927.39	948.42	942.37	959.77	944.20	950.31	949.01
20	945.85	947.93	963.56	944.12	922.29	942.27	934.92	952.57	934.94	940.52	938.44
21	957.82	960.06	977.20	956.03	921.32	953.91	948.32	963.70	947.25	953.01	950.99
22	965.68	968.73	988.65	963.92	908.65	961.93	941.80	973.97	944.13	961.34	959.80
23	967.58	969.66	988.75	965.70	911.90	963.36	959.86	972.36	957.46	962.87	960.79
24	825.58	831.52	843.75	824.37	836.85	822.58	829.16	825.42	836.04	822.51	821.08
25	855.01	859.17	874.35	853.70	857.72	851.29	854.43	854.29	860.84	850.83	849.33
26	861.71	867.74	880.98	860.48	865.27	857.92	866.39	860.44	872.17	859.62	858.58
27	867.16	873.12	888.14	865.89	860.63	863.03	870.55	866.80	876.78	865.05	864.11
28	877.13	882.69	899.74	875.81	854.07	872.61	880.51	876.98	884.39	874.30	873.22
29	870.38	873.80	890.84	869.03	860.08	866.37	867.29	870.76	873.30	865.86	864.37
30	881.75	886.71	902.42	880.43	876.63	877.14	883.46	878.77	888.08	879.44	878.43
31	899.59	903.60	921.23	898.23	876.52	895.09	897.86	900.07	902.58	896.47	895.09
32	917.09	921.96	943.02	915.69	846.17	912.73	917.30	918.58	902.87	902.16	871.90
33	797.66	801.23	818.69	796.28	806.68	793.86	797.89	797.67	802.48	793.66	791.89

34	813.51	816.41	836.26	811.95	815.75	806.53	813.38	812.20	816.89	810.57	808.72
35	817.77	820.46	842.44	816.15	809.32	812.44	816.85	816.76	820.69	814.95	813.06
36	840.06	844.82	861.85	838.75	837.38	836.15	842.83	838.95	846.06	837.10	835.80
37	851.88	858.14	876.17	850.61	838.57	848.06	854.78	851.96	857.60	850.56	849.54
38	826.29	856.89	876.66	851.10	842.72	847.91	855.46	851.22	857.72	850.22	848.81
39	735.10	737.53	761.15	733.63	745.80	729.90	743.67	739.76	748.45	734.45	732.67
40	730.63	734.12	766.82	729.43	747.96	726.61	751.75	738.28	755.89	733.84	732.32
41	759.62	761.48	774.97	758.35	768.47	755.69	759.06	761.74	762.80	756.56	754.88
42	781.94	783.32	799.32	780.42	785.09	776.62	780.44	783.62	783.43	780.43	778.57
43	789.05	790.02	808.46	787.41	781.70	773.31	785.79	790.47	789.34	787.82	785.88
44	793.28	795.21	814.78	791.62	775.51	786.87	789.90	794.80	793.57	792.34	790.46
45	803.02	803.44	821.79	801.29	794.79	796.34	804.93	799.37	805.06	802.36	800.42
46	793.28	794.01	814.78	791.61	775.50	786.86	789.88	794.81	793.57	792.33	790.45
47	795.77	796.42	818.80	794.08	766.78	789.10	792.09	797.44	795.84	795.15	793.30
48	797.02	797.59	821.82	795.31	756.93	790.25	793.49	799.01	797.22	796.47	794.66
49	797.86	798.39	824.11	796.15	746.26	791.01	794.30	800.18	798.04	797.41	795.61
50	798.38	798.88	826.26	796.66	735.56	791.48	795.03	801.07	798.72	797.80	796.19
51	793.66	792.24	810.28	792.04	793.40	789.03	785.03	796.90	787.21	788.03	785.33
52	812.75	811.62	831.27	810.99	805.95	807.15	804.27	815.62	805.55	808.90	806.30
53	830.73	829.24	850.82	828.85	809.56	824.28	826.20	828.74	824.13	827.92	825.35
54	829.96	829.10	850.29	828.09	815.81	823.62	821.90	832.15	822.28	827.54	825.01
55	846.08	845.17	867.96	844.11	815.43	839.28	845.04	842.90	840.44	843.84	841.83
56	717.38	722.34	732.31	716.29	728.58	714.98	717.36	721.10	723.04	713.82	711.61
57	777.88	783.83	794.48	776.59	787.12	773.73	781.52	777.71	783.87	778.32	776.56
58	791.68	796.83	811.51	790.20	792.89	786.06	791.53	792.71	795.28	792.78	790.87
59	802.56	807.40	824.66	800.96	789.70	795.90	804.11	799.72	806.13	804.32	802.46
60	823.06	829.29	840.86	823.06	823.84	818.44	826.74	823.28	828.73	824.36	823.02
61	836.36	844.25	856.90	835.10	827.18	831.83	839.61	837.95	841.63	839.69	838.69
62	846.58	853.93	869.33	845.22	818.80	841.17	851.53	845.11	851.42	850.14	848.96
63	843.82	851.80	865.86	842.52	824.23	838.89	846.33	845.18	848.31	847.48	846.65
64	852.37	860.04	877.56	850.93	802.31	846.88	860.11	851.23	856.56	856.38	855.07
65	849.51	857.27	873.72	848.19	818.66	844.35	851.43	851.48	853.81	853.14	852.32
66	760.63	767.74	780.59	759.54	776.41	758.27	767.80	762.14	774.11	758.43	757.06
67	790.05	797.92	820.64	789.12	806.49	787.61	805.89	794.86	813.53	790.19	789.33
68	804.65	812.14	825.96	803.53	814.44	801.42	811.81	804.97	817.92	803.73	802.80
69	822.78	830.89	845.79	821.77	820.32	819.90	832.87	823.18	837.32	822.03	821.24
70	828.04	835.07	853.08	826.76	809.87	823.70	836.69	825.54	840.34	827.32	826.16

71	830.21	838.14	856.70	828.93	799.35	826.35	838.30	829.09	842.43	829.78	828.76
72	824.76	837.13	850.01	827.20	813.00	825.13	837.81	828.71	842.68	828.74	828.12

[1] Serial No according to Table S1.

Table S3. Computed PAs calculated (in kJ/mol) at CAM-B3LYP/Def2-TZVP/epc/2s2p2d. where each epc is indicated in the table.

Serial No[1]	DFT (CAM-B3LYP/def2-TZVP	No-epc	epc17-2	epc19
1	889.01	776.10	854.90	856.99
2	891.45	776.56	855.49	856.43
3	927.41	812.35	891.34	892.3
4	934.40	819.34	898.30	899.23
5	947.86	832.61	911.61	912.41
6	954.23	838.83	917.86	918.61
7	957.88	842.42	921.45	922.16
8	960.27	844.78	923.81	924.50
9	958.30	842.78	921.81	922.43
10	958.25	843.00	921.98	922.73
11	959.53	844.11	923.11	923.80
12	960.21	844.79	923.80	924.48
13	960.60	845.18	924.18	924.86
14	960.89	845.46	924.47	925.15
15	961.09	845.66	924.67	925.35
16	963.29	846.51	925.57	925.56
17	975.39	858.46	937.53	937.37
18	985.13	868.08	947.18	946.95
19	986.40	869.34	948.42	948.11
20	981.26	863.18	942.27	941.54
21	992.95	874.80	953.91	953.04
22	1000.8	882.84	961.93	961.12
23	1002.50	884.25	963.36	962.39
24	852.42	742.64	822.58	828.49
25	881.73	771.23	851.29	856.86
26	888.67	777.86	857.92	863.31
27	893.89	782.94	863.03	868.36
28	903.61	792.47	872.61	877.87
29	897.41	786.15	866.37	871.54
30	908.46	796.93	877.14	882.11
31	926.97	814.76	895.09	899.74

32	944.68	832.44	912.73	917.42
33	822.90	714.01	793.86	800.47
34	835.90	726.62	806.53	812.94
35	841.88	732.52	812.44	818.81
36	866.42	756.09	836.15	842.09
37	878.11	768.12	848.06	853.86
38	878.42	767.82	847.91	853.69
39	757.96	650.19	729.90	736.32
40	752.73	647.70	726.61	734.22
41	783.87	676.02	755.69	762.94
42	805.46	696.85	776.62	783.37
43	802.28	693.53	773.31	780.00
44	815.91	707.07	786.87	793.49
45	825.72	716.49	796.34	802.71
46	815.91	707.07	786.86	793.49
47	818.18	709.30	789.10	795.71
48	819.35	710.45	790.25	796.85
49	820.12	711.21	791.01	797.59
50	820.59	711.67	791.48	798.06
51	819.27	709.15	789.03	795.13
52	837.67	727.25	807.15	812.92
53	855.30	744.31	824.28	829.76
54	854.38	743.70	823.62	829.10
55	870.37	759.31	839.28	844.50
56	742.53	635.76	714.98	722.23
57	802.80	694.20	773.73	780.02
58	815.40	706.50	786.06	792.19
59	825.44	716.29	795.90	801.89
60	848.55	738.70	818.44	824.22
61	861.66	752.23	831.83	837.43
62	871.09	761.54	841.17	846.68
63	868.86	759.27	838.89	844.41
64	876.89	767.28	846.88	852.26
65	874.29	764.73	844.35	849.86

66	786.45	678.58	758.27	765.36
67	815.03	708.27	787.61	794.59
68	830.96	721.49	801.42	772.69
69	849.65	739.94	819.90	826.07
70	853.63	743.72	823.70	829.79
71	855.59	745.66	826.35	832.43
72	854.96	745.15	825.13	831.24

[1] Serial No according to Table S1.

Table S4. Computed PAs (in kJ/mol) calculated at CAM-B3LYP/electronic basis set/epc17-2/2s2p2d. where each electronic basis set is indicated in the table.

Serial No[1]	def2-SVP	def2-TZVP	def2-QZVP
1	884.21	854.88	852.77
2	878.57	855.47	855.6
3	913.34	891.32	891.41
4	919.10	898.28	898.09
5	930.62	911.59	912.18
6	933.73	917.84	918.96
7	936.67	921.43	922.43
10	939.16	921.96	923.06
16	939.49	925.55	927.12
18	959.00	947.16	949.67
19	960.42	948.41	951.18
20	950.77	942.26	944.76
21	962.14	953.89	956.74
24	842.35	822.56	824.38
25	866.47	851.27	854.05
26	877.46	857.91	859.92
27	880.10	863.02	865.49
28	889.26	872.60	875.33
29	877.62	866.36	870.72
30	891.98	877.12	880.12
36	850.14	836.14	839.53
37	859.52	848.05	851.96
38	860.57	847.89	851.71
39	750.09	729.88	731.12
40	751.73	726.60	727.99
41	777.59	755.68	756.08
42	795.41	776.6	778.33
43	799.65	773.3	784.99
44	802.88	786.85	788.86
45	812.61	796.32	798.62
46	802.88	786.85	788.86

47	804.69	789.09	791.12
48	805.60	790.24	792.27
51	802.81	789.02	791.84
52	818.95	807.14	810.71
53	835.00	824.27	828.10
54	833.86	823.61	827.78
56	728.11	714.96	717.34
57	786.25	773.71	776.78
58	796.75	786.05	789.45
59	805.64	795.88	799.44
60	830.94	818.43	821.47

[1] Serial No according to Table S1.

Table S5. Computed PAs calculated at CAM-B3LYP/Def2-TZVP/epc17-2/nuclear basis set. where each nuclear basis set is indicated in the table.

Serial No[1]	2s2p2d	4s4p4d	6s6p6d	8s8p8d	10s10p10d
1	854.90	856.74	856.74	856.79	856.77
2	855.49	857.49	857.49	857.54	857.53
3	891.34	893.36	893.37	893.41	893.40
4	898.30	900.32	900.32	900.37	900.36
5	911.61	913.65	913.65	913.7	913.68
6	917.86	919.91	919.91	919.96	919.95
7	921.45	923.51	923.51	923.56	923.54
8	923.81	925.87	925.87	925.92	925.91
9	921.81	923.87	923.87	923.92	923.91
10	921.98	924.01	924.01	924.06	924.05
11	923.11	925.16	925.17	925.21	925.20
12	923.80	925.85	925.85	925.89	925.88
13	924.18	926.23	926.53	926.29	926.27
14	924.47	926.52	926.52	926.57	926.56
15	924.67	926.72	926.72	926.77	926.76
16	925.57	927.74	927.74	927.79	927.78
17	937.53	939.72	939.72	939.77	939.75
18	947.18	949.38	949.38	949.43	949.41
19	948.42	950.62	950.63	950.67	950.65
20	942.27	944.57	944.57	944.62	944.61
21	953.91	956.21	956.21	956.25	956.24
22	961.93	964.20	964.20	964.25	964.24
23	963.36	965.67	965.67	965.71	965.70
24	822.58	824.24	824.26	824.32	824.30
25	851.29	853.01	853.03	853.09	853.07
26	857.92	859.68	859.69	859.75	859.73
27	863.03	864.80	864.81	864.87	864.85
28	872.61	874.40	874.41	874.47	874.45
29	866.37	868.16	868.18	868.24	868.22
30	877.14	878.95	878.97	879.03	879.01
31	895.09	896.97	896.98	897.04	897.03

32	912.73	914.61	914.63	914.69	914.67
33	793.86	795.47	795.48	795.54	795.52
34	806.53	808.16	808.18	808.24	808.22
35	812.44	814.08	814.10	814.16	814.14
36	836.15	837.88	837.89	837.95	837.93
37	848.06	849.76	849.77	849.83	849.81
38	847.91	849.66	849.67	849.73	849.71
39	729.90	731.41	731.43	731.48	731.47
40	726.61	727.92	727.92	727.98	727.96
41	755.69	757.22	757.23	757.29	757.27
42	776.62	778.20	778.21	778.27	778.25
43	773.31	774.91	774.92	774.98	774.96
44	786.87	788.46	788.48	788.54	788.52
45	796.34	797.96	797.98	798.04	798.03
46	786.86	788.46	788.48	788.53	788.52
47	789.10	790.71	790.72	790.78	790.76
48	790.25	791.86	791.87	791.93	791.91
49	791.01	792.61	792.63	792.69	792.66
50	791.48	793.08	793.09	793.15	793.14
51	789.03	790.74	790.75	790.81	790.79
52	807.15	808.88	808.89	808.95	808.93
53	824.28	826.06	826.07	826.13	826.11
54	823.62	825.37	825.38	825.44	825.42
55	839.28	841.07	841.08	841.14	841.12
56	714.98	716.42	716.43	716.49	716.47
57	773.73	775.31	775.32	775.37	775.35
58	786.06	787.67	787.68	787.73	787.72
59	795.90	797.52	797.53	797.58	797.56
60	818.44	820.12	820.14	820.20	820.17
61	831.83	833.47	833.48	833.54	833.52
62	841.17	842.82	842.83	842.89	842.87
63	838.89	840.55	840.56	840.61	840.60
64	846.88	848.53	848.54	848.60	848.58
65	844.35	846.00	846.01	846.06	846.05

66	758.27	759.80	759.81	759.87	759.85
67	787.61	789.05	789.06	789.12	789.10
68	801.42	803.07	803.09	803.14	803.13
69	819.90	821.57	821.59	821.65	821.63
70	823.70	825.39	825.4	825.46	825.44
71	826.35	828.04	827.37	828.11	827.43
72	825.13	826.81	826.82	839.06	826.86

[1] Serial No according to Table S1.

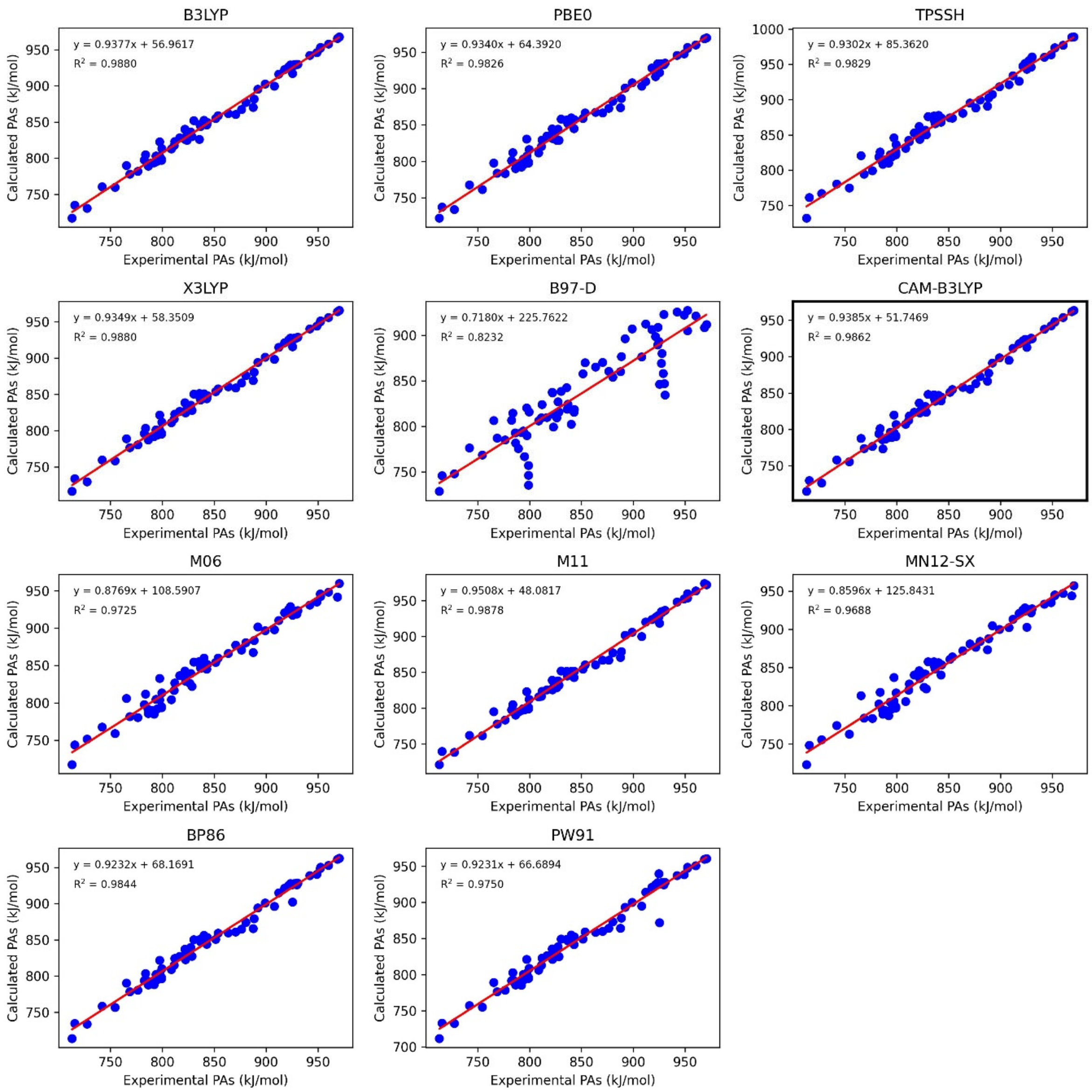

Figure S1. Experimental and computed PAs for different functionals with linear regression (red line).

.

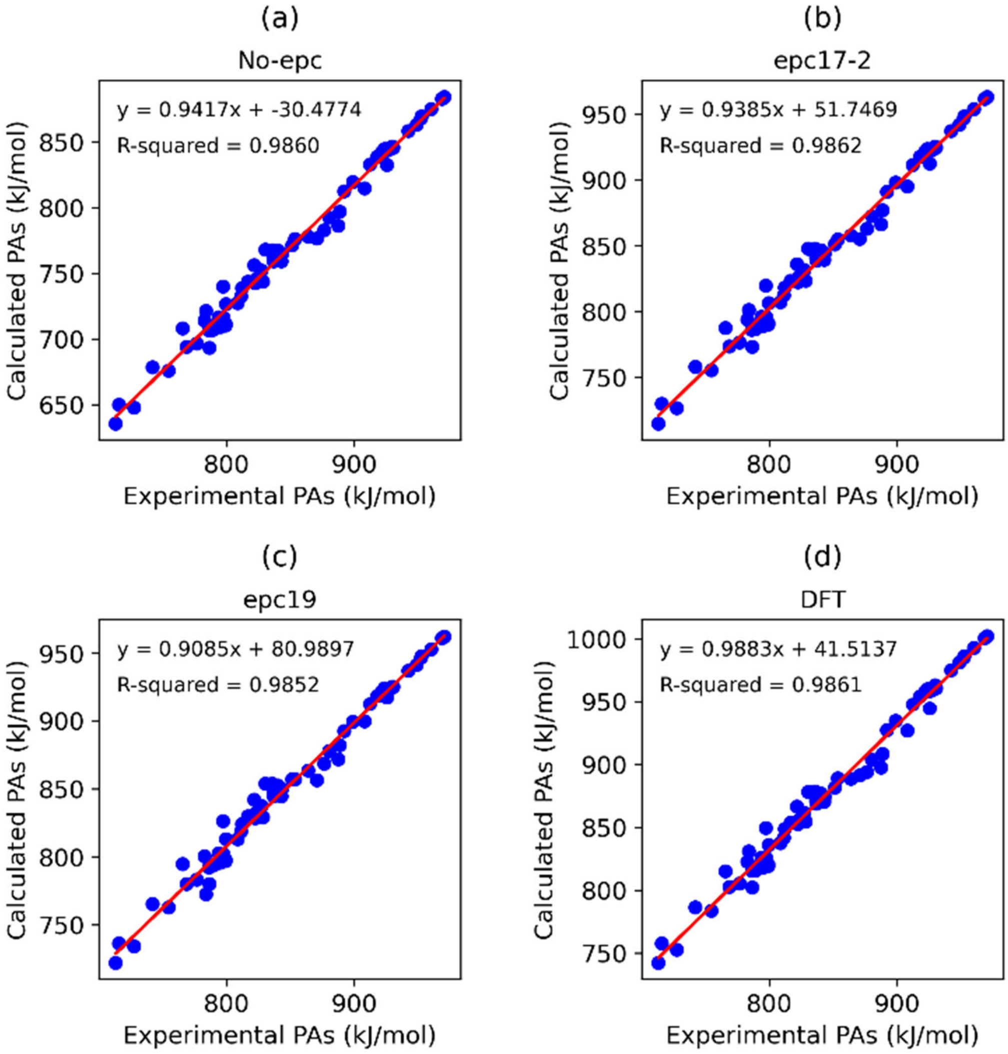

Figure S2. Experimental and computed PAs for a) No-epc, b) epc17-2, c) epc19 together with linear regression (red line).

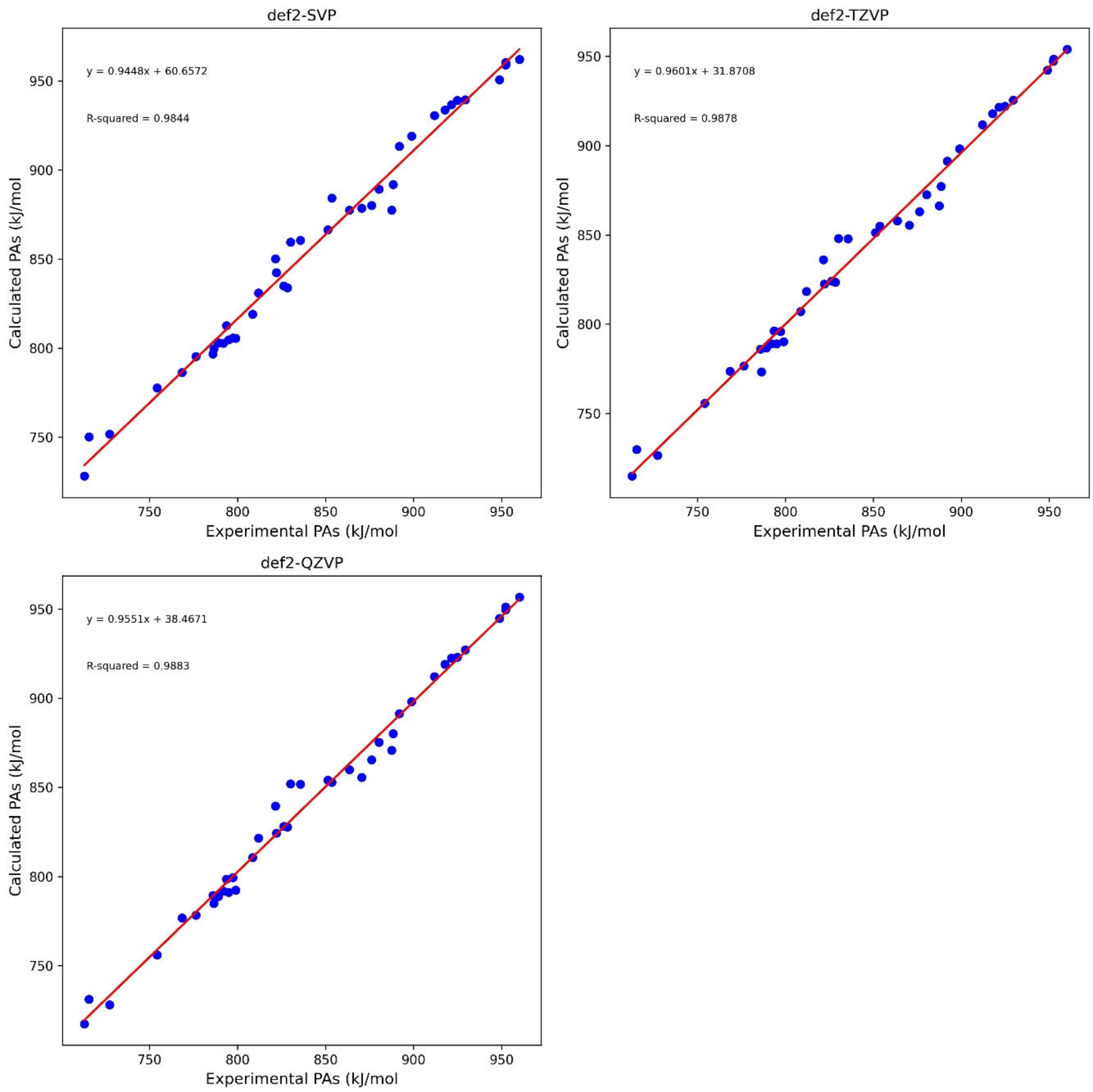

Figure S3. Combined result for experimental vs predicted PAs for different electronic basis sets together with linear regression (red line).

.

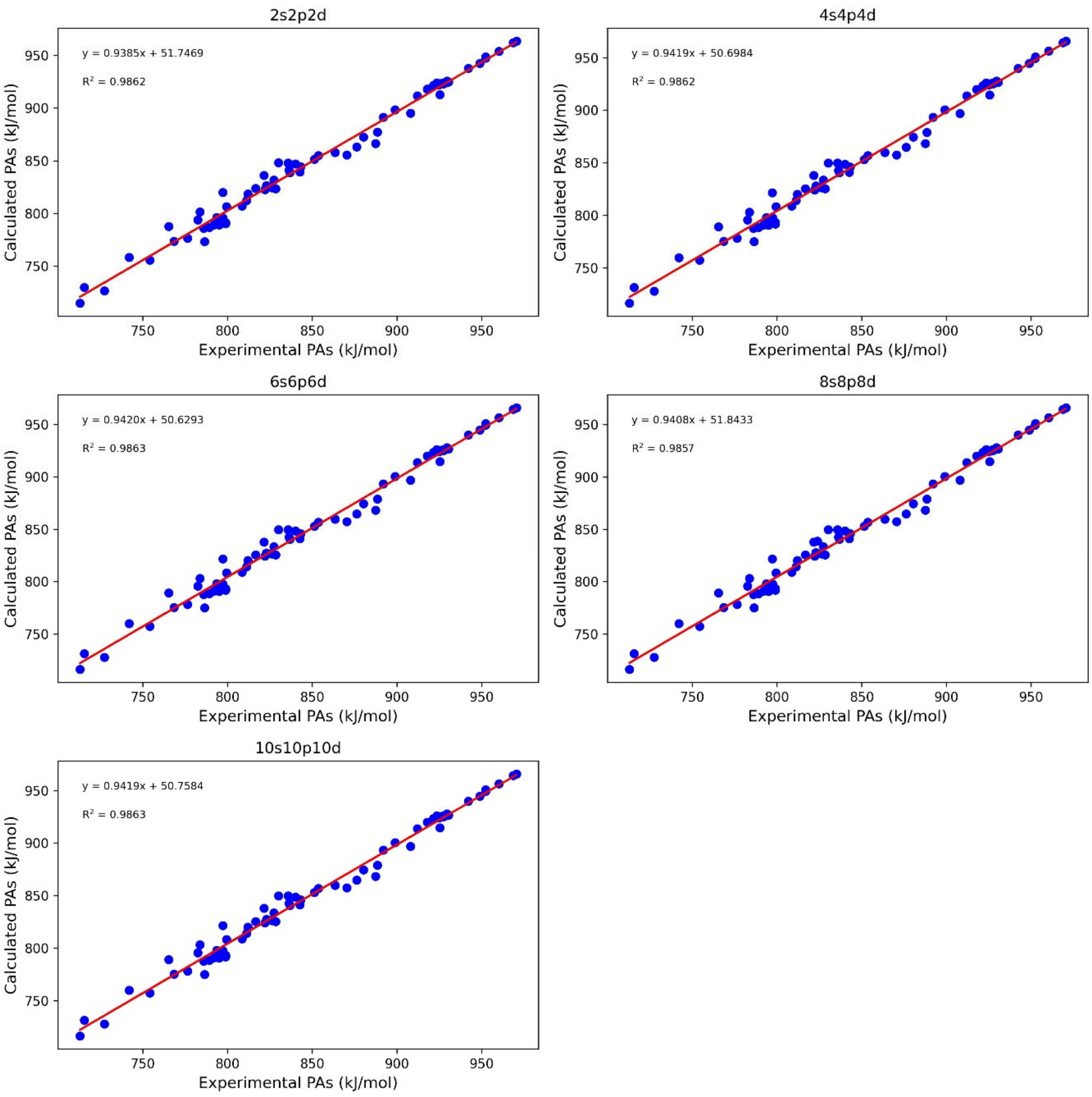

Figure S4. Combined result for experimental vs predicted Pas for different nuclear basis sets linear regression (red line).

.

References

1. Hunter, E.P.L., S. G, *NIST Proton Affinity Database, Standard Referenz Database Number 69, National Institute of Standards and Technology, Nov. 1998.* 1998.
